# Overexpression of AMPKγ2 increases AMPK signaling to augment human T cell metabolism and function

**DOI:** 10.1016/j.jbc.2023.105488

**Published:** 2023-11-22

**Authors:** Erica L. Braverman, Margaret A. McQuaid, Herbert Schuler, Mengtao Qin, Sophia Hani, Keli Hippen, Darlene A. Monlish, Andrea K. Dobbs, Manda J. Ramsey, Felicia Kemp, Christopher Wittmann, Archana Ramgopal, Harrison Brown, Bruce Blazar, Craig A. Byersdorfer

**Affiliations:** 1 Division of Blood and Marrow Transplant and Cellular Therapies, Department of Pediatrics, University of Pittsburgh School of Medicine, Pittsburgh, Pennsylvania, USA; 2 School of Medicine, Tsinghua University, Beijing, China; 3 Division of Blood and Marrow Transplantation, Department of Pediatrics, University of Minnesota, Minneapolis, Minnesota, USA

**Keywords:** immunometabolism, AMP-activated protein kinase, AMPK, oxidative metabolism, glycolysis, Th1, Th2, spare respiratory capacity, glucose restriction, Treg, memory T cells

## Abstract

Cellular therapies are currently employed to treat a variety of disease processes. For T cell–based therapies, success often relies on the metabolic fitness of the T cell product, where cells with enhanced metabolic capacity demonstrate improved *in vivo* efficacy. AMP-activated protein kinase (AMPK) is a cellular energy sensor which combines environmental signals with cellular energy status to enforce efficient and flexible metabolic programming. We hypothesized that increasing AMPK activity in human T cells would augment their oxidative capacity, creating an ideal product for adoptive cellular therapies. Lentiviral transduction of the regulatory AMPKγ2 subunit stably enhanced intrinsic AMPK signaling and promoted mitochondrial respiration with increased basal oxygen consumption rates, higher maximal oxygen consumption rate, and augmented spare respiratory capacity. These changes were accompanied by increased proliferation and inflammatory cytokine production, particularly within restricted glucose environments. Introduction of AMPKγ2 into bulk CD4 T cells decreased RNA expression of canonical Th2 genes, including the cytokines interleukin (IL)-4 and IL-5, while introduction of AMPKγ2 into individual Th subsets universally favored proinflammatory cytokine production and a downregulation of IL-4 production in Th2 cells. When AMPKγ2 was overexpressed in regulatory T cells, both *in vitro* proliferation and suppressive capacity increased. Together, these data suggest that augmenting intrinsic AMPK signaling *via* overexpression of AMPKγ2 can improve the expansion and functional potential of human T cells for use in a variety of adoptive cellular therapies.

Recently, a clear link has emerged between T cell metabolism and T cell fate and function (1, 2, 3). In particular, adoptive cellular therapies for cancer, including chimeric antigen receptor (CAR) T cells and tumor-infiltrating lymphocytes, demonstrate reduced efficacy when faced with the constraints of the *in vivo* metabolic environment, which often correlates with decreased persistence of the transferred cells (4, 5, 6). In contrast, adoptive T cell therapies with increased oxidative capacity and preserved mitochondrial function experience enhanced performance both *in vitro* and during subsequent transfer *in vivo* (7, 8). For this reason, extensive efforts have sought to improve the metabolic capacity of T cells, with the goal of enhancing metabolic plasticity and long-term survival *in vivo*. Several *in vitro* interventions to reduce T cell differentiation and minimize metabolic exhaustion have been reported, including nutrient restriction, inhibition of protein synthesis, expansion in the presence of exogenous cytokines (*e.g.*, interleukin (IL)-7 or IL-15), blockade of mitochondrial fission, promotion of mitochondrial fusion, and upregulation of mitochondrial biogenesis (9, 10, 11, 12, 13, 14, 15). While various approaches can successfully enhance oxidative metabolism, a major drawback to these methods is a sharp reduction in T cell proliferation, limiting their clinical applicability. In addition, numerous pharmaceutical interventions are operational only *in vitro*, which may not provide a long-term functional benefit *in vivo*. For this reason, the search continues to identify and develop durable and effective approaches to optimizing T cell metabolic capacity while preserving proliferation and limiting *in vitro* differentiation. Modulating immune cell metabolism through manipulation of the cellular energy sensor, AMP-activated protein kinase (AMPK), offers the potential to achieve this goal.

AMPK is traditionally recognized for detecting decreasing intracellular energy stores through a rise in AMP and concomitant decrease in ATP levels. When the overall AMP/ATP ratio increases, AMPK becomes activated and then phosphorylates downstream targets to block anabolic growth, promote catabolic metabolism, and enhance mitochondrial efficiency. AMPK achieves this mitochondrial efficiency by driving mitophagy to clear dysfunctional mitochondria, stimulating mitochondrial biogenesis to create new mitochondria, and facilitating mitochondrial fusion to promote more efficient mitochondria (16). Alongside these efforts, additional AMPK-driven pathways scavenge reactive oxygen species and enhance fatty acid oxidation. By incorporating environmental signals, AMPK can specifically drive metabolic programming matched to the nutritional milieu, promoting optimal function (17). When energy stores are low, AMPK’s role in anabolic growth blockade centers on its negative regulation of mammalian target of rapamycin (mTOR) signaling (18) and blockade of fatty acid and cholesterol synthesis (16, 19, 20, 21, 22, 23, 24, 25). Together, these efforts create more efficient cells with higher capacity to withstand cellular stress, a combination which can facilitate *in vivo* health and may potentiate antitumor activity of adoptive cellular therapies. In support of AMPK’s role in anticancer immune responses, global knock out of AMPK in three different mouse tumor models significantly decreased tumor cell killing (26), while driving AMPK activity benefits antitumor responses when combined with checkpoint blockade (27). Together, these data highlight AMPK’s responsibility in controlling pathways critical for maximal T cell function *in vivo*.

AMPK is a heterotrimeric protein complex consisting of α, β, and γ subunits, with each subunit having multiple isoforms (α1, α2, β1, β2, and γ1, γ2, and γ3) (28). The α subunit, which houses the kinase activity, is active when phosphorylated on amino acid Thr172 by upstream activators of AMPK. These activators include liver kinase B-1 and calcium influx, which signals through calcium/calmodulin-dependent protein kinase kina to drive AMPK activation following T cell receptor (TCR) stimulation (29). Once phosphorylated, the α domain of AMPK phosphorylates downstream targets, including Unc51-like kinase 1 (ULK1), acetyl-CoA carboxylase, and peroxisome proliferator–activated receptor-gamma coactivator-1 α (PGC1α) (25, 30, 31, 32). In counterbalance, phosphatases, including the protein phosphatase 2A heterotrimer, dephosphorylate AMPK to downregulate its activity (33). Importantly, AMPKα dephosphorylation is hindered by actions of the regulatory γ subunit, which preserves kinase activity and provides additional allosteric activation of AMPK in response to AMP levels (34). Of note, the different gamma isoforms (γ1, γ2, γ3) mediate differential effects, with AMPKγ2 allowing for more prolonged AMPK signaling *via* both improved protection of the AMPKα domain (thought to be related to its longer N terminus) and a higher sensitivity for sensing ADP in addition to AMP (28).

Many pharmacologic agents increase AMPK activity, metformin being the most well-known. However, many of these interventions, including metformin, increase intracellular AMP/ATP ratios by impairing mitochondrial respiration, in effect starving the cell. One downside of this approach is the creation of mitochondrial dysfunction, which is counterproductive to generating an optimal T cell response. This problem is shared with other agonists, including 5-aminoimidazole-4-carboxamide ribonucleotide, which functions as an AMP mimetic. In addition to being nonspecific (reviewed in (17)), mimetic treatment also relies on simulating cellular starvation, a process which downregulates critical cellular pathways, including cell growth and protein synthesis, which are needed to generate effective immune responses. Altogether, these studies suggest that forcing cells to increase AMPK signaling through perceived or actual nutrient starvation may hinder immune responses more than they help. In contrast, a method to selectively increase AMPK activity in T cells, without driving starvation-mediated growth blockade and with the possibility of *in vivo* durability, would be ideal.

To increase AMPK signaling in primary human T cells, we elected to overexpress the regulatory subunit AMPKγ2, a process we hypothesized would enhance and prolong kinase activity by prolonging the phosphorylation of Thr172. Here, we demonstrate that overexpression of AMPKγ2 stably increases AMPK activity in human T cells, leading to enhanced mitochondrial mass, heightened spare respiratory capacity, and improved metabolic efficiency, alongside increased *in vitro* expansion. In addition, introduction of AMPKγ2 universally favored proinflammatory cytokine production in transduced bulk T cells, with a notable reduction in the generation of a Th2 phenotype. As such, AMPKγ2 overexpression, working through multiple pathways, endows T cells with characteristics ideal for subsequent use in adoptive cellular therapies.

## Results

### AMPKγ2 overexpression increases AMPK activity in primary human T cells

**Figure 1:**
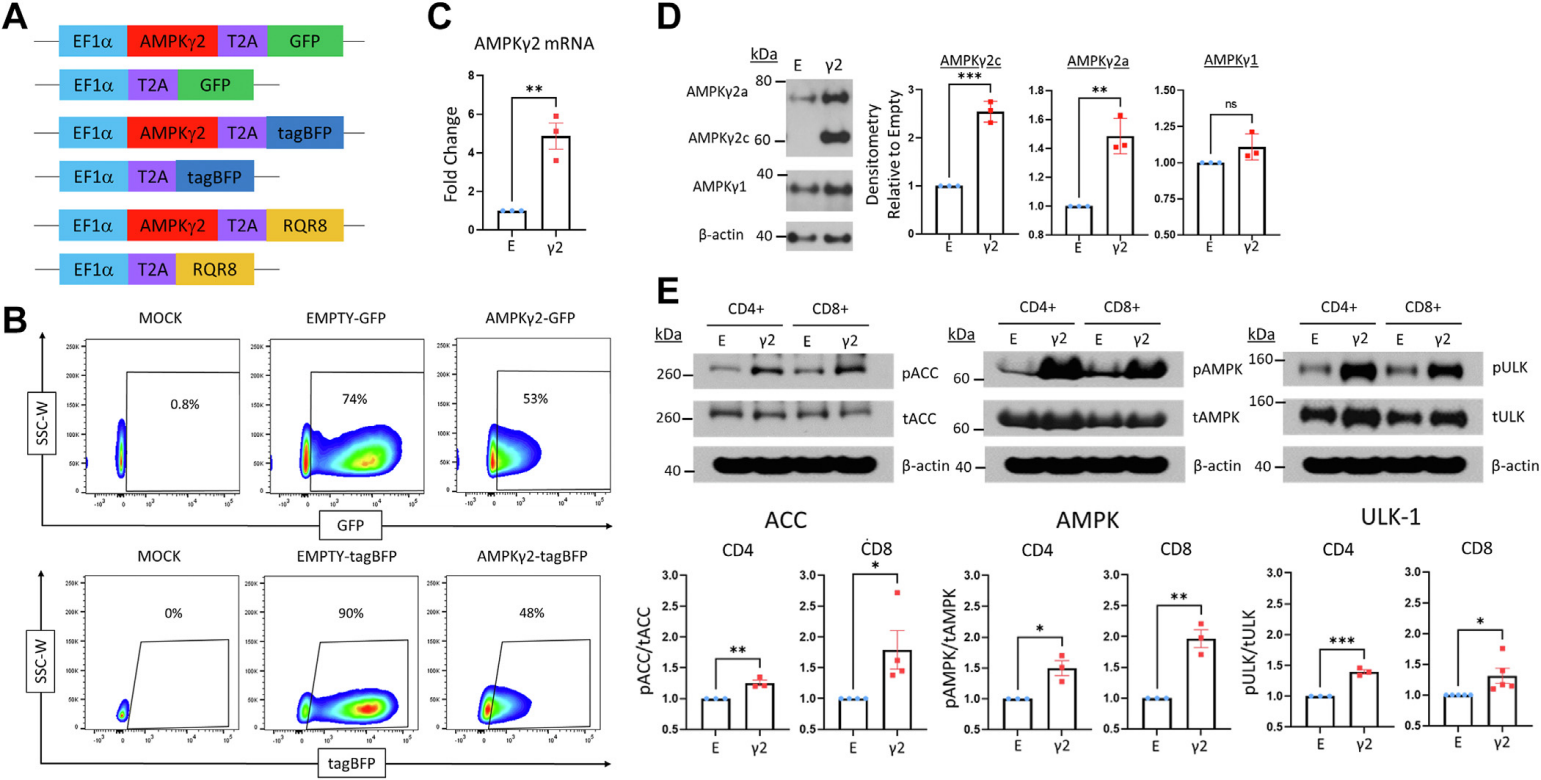
**AMPKγ2 overexpression increases AMPK activity in human T cells.***A*, schematic of AMPKγ2 and tag only “empty” or “empty vector” control vectors with an EF1α promoter and either GFP, tagBFP, or RQR8 expression tag. *B*–*D*, primary human T cells were mock transduced or transduced with AMPKγ2 or empty plasmids. Expression was verified by flow cytometry for GFP or tagBFP tag (*B*), fold change in AMPKγ2 mRNA expression using qRT-PCR (*C*), or immunoblot to detect protein expression of AMPKγ2c (the transduced isoform), as well as native AMPKγ2a and AMPKγ1 (*D*). Blot was cut at ∼50 kDa, after which top half was developed for AMPKγ2 and bottom half probed for AMPKγ1, then stripped and reprobed for β-actin. The top of this same blot was subsequently stripped and reprobed for PGC1α, optic atrophy gene 1, and mitofusin 1 (see Fig. 2). *E*, human T cells were transduced with AMPKγ2- *versus* empty constructs, cells lysates collected on days 9 to 12, and phosphorylation of AMPKα on Thr172 (to detect AMPK activation), ACC Ser79, and ULK-1 Ser555 measured by immunoblot. The same β-actin control blot is displayed multiple times for purposes of comparison. Accompanying densitometry was measured on immunoblots from multiple donors using ImageJ software, followed by normalization of AMPKγ2-transduced cells in each sample to empty controls. All data represent three or more independent human donor samples. Protein size markers are shown in kDa. ∗*p* < 0.05, ∗∗*p* < 0.01, and ∗∗∗*p* < 0.001 by paired Student’s *t* test. ACC, acetyl-CoA carboxylase; AMPK, AMP-activated protein kinase; EF1α, elongation factor 1 alpha; PGC1α, peroxisome proliferator–activated receptor-gamma coactivator-1 α; ULK1, Unc51-like kinase 1.

To increase AMPK signaling in human T cells, we focused our attention on modulating expression of the regulatory subunit, AMPKγ2. We hypothesized that overexpression of AMPKγ2 would enhance AMPK activity in T cells, both through earlier AMPK activation due to its sensitivity to ADP (35) as well as through prolonged activation once AMPK had been phosphorylated due to its longer N terminus (36). To test this idea, primary human T cells were stimulated with CD3/CD28 Dynabeads, followed by transduction with lentiviral constructs containing an AMPKγ2 sequence downstream from an elongation factor 1 alpha promoter and upstream from either a fluorescent or surface marker tag (37) separated by a T2A linker (Fig. 1*A*). To note, AMPKγ2 exists in five defined isoforms which differ in the length and composition of their N terminus (https://www.ncbi.nlm.nih.gov/gene/51422) but with no currently described differences in the functions of these various γ2 isoforms. For these experiments, we elected to overexpress isoform C, which has a slightly truncated N terminus as compared to the more highly produced isoform A. This selection incidentally allowed easy identification of transduced cells by Western blot. Control cells were transduced with “empty” constructs containing only the T2A sequence and the matched tag. On day five posttransduction, tag expression revealed efficient transduction of both plasmids (Figs. 1*B* and S1) and RNA harvested on day 9 revealed a 5-fold increase in AMPKγ2 levels compared to empty controls (Fig. 1*C*). Due to its smaller size, our transduced AMPKγ2 protein could easily be separated from the more abundant isoform A by immunoblot analysis, which confirmed increased isoform C expression in AMPKγ2-transduced cells (Fig. 1*D*). Interestingly, AMPKγ2 isoform C transduction also upregulated isoform A expression, without impacting expression of native AMPKγ1 (Fig. 1*D*). We then asked whether overexpression of the regulatory γ domain was sufficient to increase AMPK activity. Tag+ CD4+ and CD8+ T cells were flow sorted after roughly 1 week of culture and Thr172 phosphorylation of the AMPKα subunit assessed by immunoblot. Overexpression of AMPKγ2 increased phosphorylation of AMPKα in both CD4 and CD8 T cells, with AMPK activity further confirmed through phosphorylation of Ser79 on acetyl-CoA carboxylase and Ser555 on ULK1 (Fig. 1*E*), two well-known AMPK targets.

### AMPKγ2 overexpression enhances metabolic capacity

**Figure 2:**
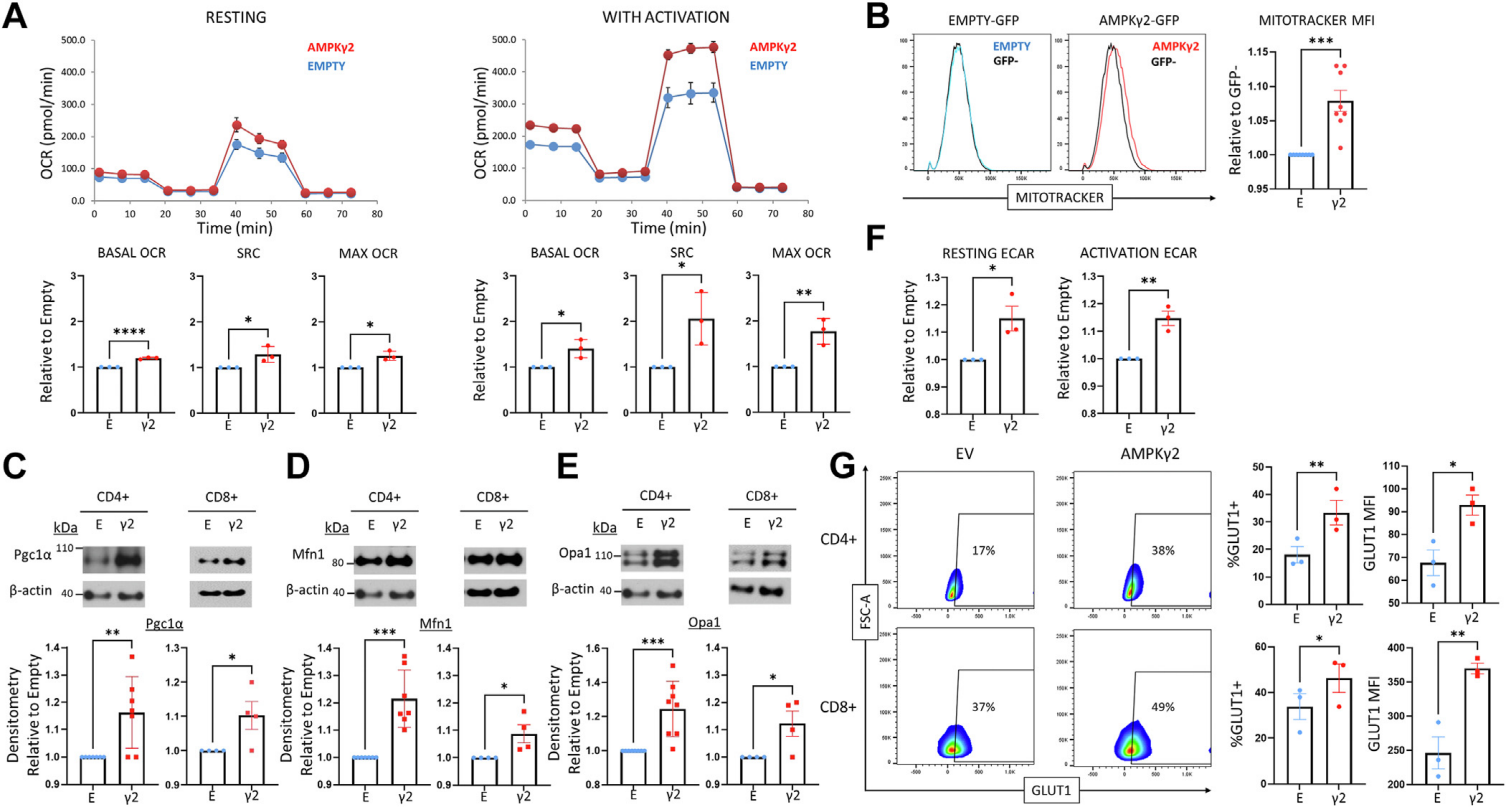
**AMPKγ2-transduced T cells enhance oxidative metabolism.***A*, human T cells were transduced with AMPKγ2 or empty lentiviral vectors and expanded in interleukin-2. On day 9, a portion of cells were stimulated overnight with anti-CD3/CD28 Dynabeads and the next day both resting (*left*) and activated (*right*) cells were assessed for oxidative capacity utilizing the Seahorse metabolic analyzer. Bar graphs represent data from three individual human donors. *B*, AMPKγ2- and empty-transduced T cells were assessed for mitochondrial density utilizing MitoTracker Red (*left*), with fluorescence compared to GFPneg controls within each sample to standardize staining between groups. Differences in median fluorescence intensity (MFI) between GFP+ and GFPneg cells was normalized to the empty control (*right*). AMPKγ2- *versus* empty-transduced T cells were sorted into CD4+ and CD8+ subsets on day 9 poststimulation and expression of the transcriptional coactivator PGC1α (*C*) or mitochondrial fusion proteins MFN1 (*D*) and OPA1 (*E*) assessed by immunoblot. As noted above, blot was cut at ∼50 kDa and top half was first developed for AMPKγ2 (Fig. 1*D*), then stripped and reprobed for PGC1α, OPA1, and MFN1 as shown in (*C–E*). Bar graphs represent data from 3 to 4 human donors. *F*, extracellular acidification rates (ECAR) were assessed in day 9 resting (*left*) or 24 h activated (*right*) transduced human T cells using the Seahorse metabolic analyzer. *G*, transduced human T cells were activated overnight and stained for GLUT1 expression. Data represent the percentage (*left bar graph*) and positive population MFI (*right bar graph*) of transduced cells. Protein size markers are shown in kDa. ∗*p* < 0.05, ∗∗*p* < 0.01, ∗∗∗*p* < 0.001, and ∗∗∗∗*p* < 0.0001 by paired Student’s *t* test. AMPK, AMP-activated protein kinase; GLUT, glucose transporter; MFN1, mitofusin 1; OPA1, optic atrophy 1; PGC1α, peroxisome proliferator–activated receptor-gamma coactivator-1 α.

We next sought to study the metabolic effects driven by AMPKγ2 overexpression. AMPKγ2- or empty-transduced cells were expanded *in vitro* in IL-2 through day 9, then placed into a Seahorse metabolic analyzer to measure basal oxygen consumption rates (OCRs), maximal respiration, and spare respiratory capacity (SRC). AMPKγ2-transduced cells modestly increased all three parameters compared to Empty-transduced controls (Fig. 2*A*). To determine whether this improvement was maintained with TCR activation, a subset of day 9 cells were stimulated overnight with anti-CD3/CD28 Dynabeads followed by subsequent analysis of metabolic activity. Following TCR stimulation, increases in basal OCR, maximal OCR, and SRC were even more pronounced in AMPKγ2-transduced cells (Fig. 2*A*). To investigate the etiology of this increased respiratory capacity, mitochondrial density was assessed using MitoTracker Red, which showed a reproducible and statistically significant increase in mitochondrial mass in AMPKγ2-transduced cells (Fig. 2*B*). To examine pathways contributing to this result, we quantitated expression of PGC1α, a known transcriptional coactivator of mitochondrial biogenesis (30) in our transduced cells. With increased AMPK activity, PGC1α becomes phosphorylated, translocates to the nucleus, and induces expression of multiple mitochondrial genes including PGC1α itself, as well as mitofusin-1 and optic atrophy gene 1, two mediators of mitochondrial fusion (38). Immunoblot analysis identified a significant increase in total PGC1α protein in both CD4+ and CD8+ T cells (Fig. 2*C*), in addition to increased expression of mitofusin-1 and optic atrophy gene 1 (Fig. 2, *D* and *E*) in the presence of additional AMPK signaling. Together, these data suggest that increased mitochondrial density, as well as enhanced mitochondrial fusion, contributes to the increase in oxidative capacity in AMPKγ2-transduced T cells.

AMPK is also known to promote glycolytic activity, for example by increasing glucose uptake and mediating downstream effects on phosphofructokinase and phosphorylation of 6-phosphofructo-2-kinase (39, 40). Resting human T cells transduced with AMPKγ2 increased their glycolytic activity (measured indirectly as the extracellular acidification rate (ECAR) on the Seahorse metabolic analyzer), which continued upon restimulation (Fig. 2*F*). Given the link between upregulated AMPK signaling and expression of glucose transporters (GLUTs) (41), we stained restimulated cells for surface expression of the GLUT1, noting an increase in both the percentage and median fluorescence intensity of GLUT1+ T cells (Fig. 2*G*). Together, these data suggest that AMPKγ2-overexpression reprograms both oxidative capacity and glycolytic flux (42).

### AMPK signaling boosts *in vitro* T cell proliferation

**Figure 3:**
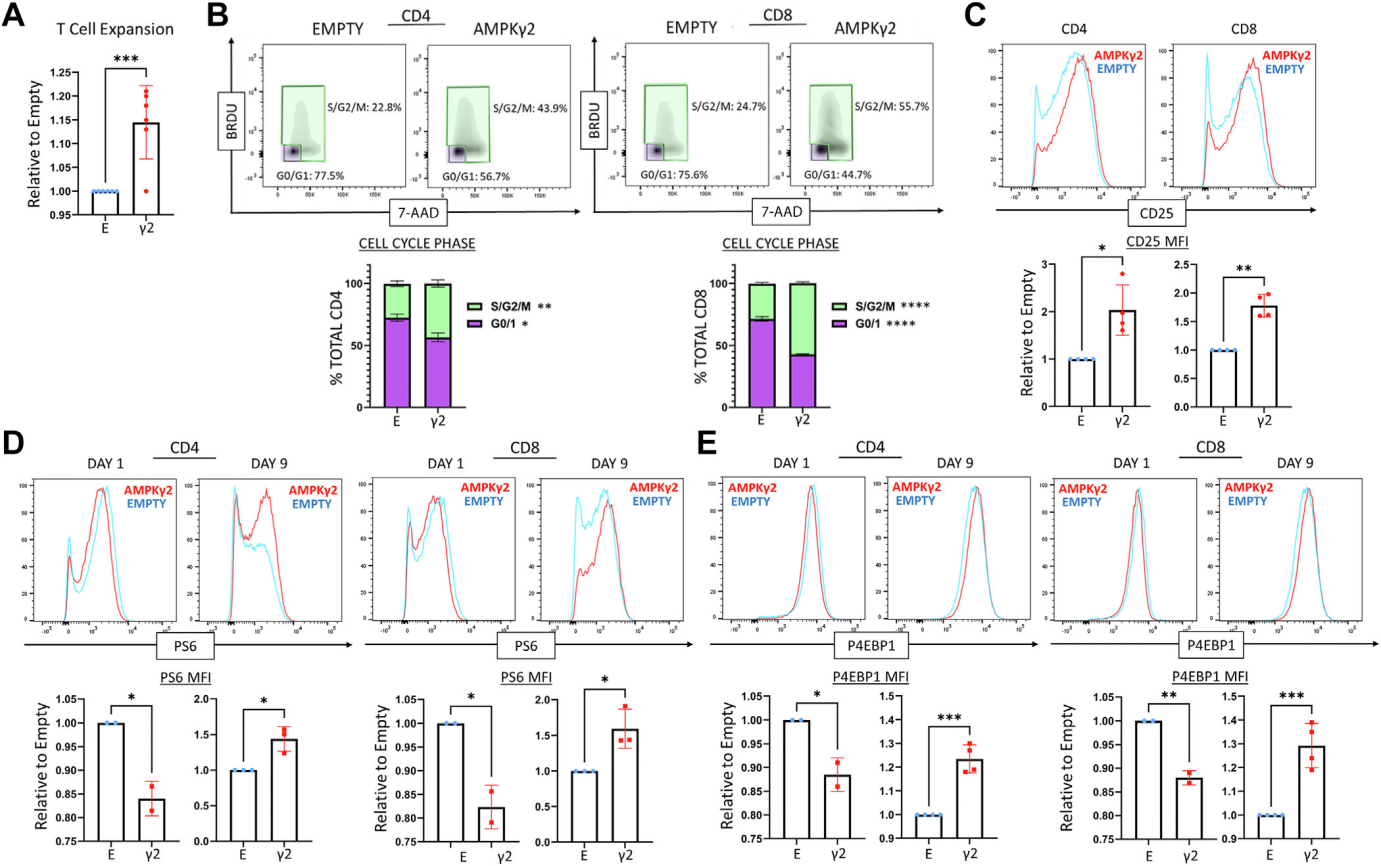
**AMPK activation increases T cell expansion, cell cycling, and mTOR activity.***A*, AMPKγ2-transduced human T cells (GFP+) were manually counted between day 5 and 7 to calculate the doubling rate per 24 h, which was then normalized to the empty-transduced control for each donor. Graph represents data from six independent human donors. *B*, AMPK- and empty-transduced T cells were expanded *in vitro* for 7 to 9 days, incubated with BrdU for the final 2 h, and costained with 7-AAD. Plots are divided into resting (G0/G1) and cycling (S, G2/M) phases. Graphs represent data from three human donors. *C*, transduced cells were harvested on day 9 of culture and stained for CD25 and MFI values compared between multiple donors (*bottom*). *D* and *E*, empty- and AMPKγ2-transduced T cells were expanded until day 9, followed by assessment of mTOR activity using antibodies against phosphorylated S6 (*D*) and 4EBP1 (*E*). Cells were assessed either at rest (day 9) or following 24 h of CD3/CD28 stimulation (day 1). The MFI for ∗P-S6 or ∗P-4EBP1 from each donor was normalized to empty-transduced controls and then compared between donors. Bar graphs represent data from four human donors. ∗*p* < 0.05, ∗∗*p* < 0.01, ∗∗∗*p* < 0.001, and ∗∗∗∗*p* < 0.0001 by paired Student’s *t* test. 7-AAD, 7-amino actinomycin D; AMPK, AMP-activated protein kinase; MFI, median fluorescence intensity; mTOR, mammalian target of rapamycin.

Many methods to increase oxidative metabolism result in restricted glycolysis, which negatively impacts cell expansion (12) and limits the clinical utility of such protocols. However, because AMPKγ2 transduction enhances both oxidative and glycolytic metabolism, it may instead boost proliferation. We first assessed cell expansion by quantitating cell numbers on days 5 and 7 of *in vitro* culture, which revealed a statistically significant increase in the doubling rate of the AMPKγ2-transduced cells per 24-h period (Fig. 3*A*). We next assessed cell cycling *via* BrdU uptake on day 9 of culture, followed by counterstaining with 7-amino actinomycin D. Both CD4 and CD8 T cells transduced with AMPKγ2 had fewer resting G0/G1 cells and more cells in the S/G2/M phases of the cell cycle (Fig. 3*B*). This increase in cell cycling was accompanied by increased expression of CD25, the α chain of the IL-2 receptor, suggesting that AMPKγ2-transduced cells may be more sensitive to extracellular levels of IL-2 (Fig. 3*C*).

Highly proliferative cells also increase signaling through mTOR to provide the necessary building blocks for continued cell division. Given the known antagonism between AMPK and mTOR pathways, we questioned whether mTOR activity was affected by AMPKγ2 overexpression. We found that mTOR signaling, as assessed by phosphorylation of downstream targets S6 and 4EBP1, increased in day 9 AMPKγ2-transduced cells, correlating with their ongoing proliferation. However, 24 h after restimulation (day 1), there was a slight but consistent decrease in mTOR signaling in AMPK-transduced cells (Fig. 3, *D* and *E*), consistent with an acute role of AMPK in decreasing mTOR signaling (43). In sum, AMPK-transduced cells maintained increased cell cycling after 1 week in culture, which correlated with increased CD25 expression and a relative increase in mTOR signaling.

### Increased AMPK signaling does not accelerate exhaustion

**Figure 4:**
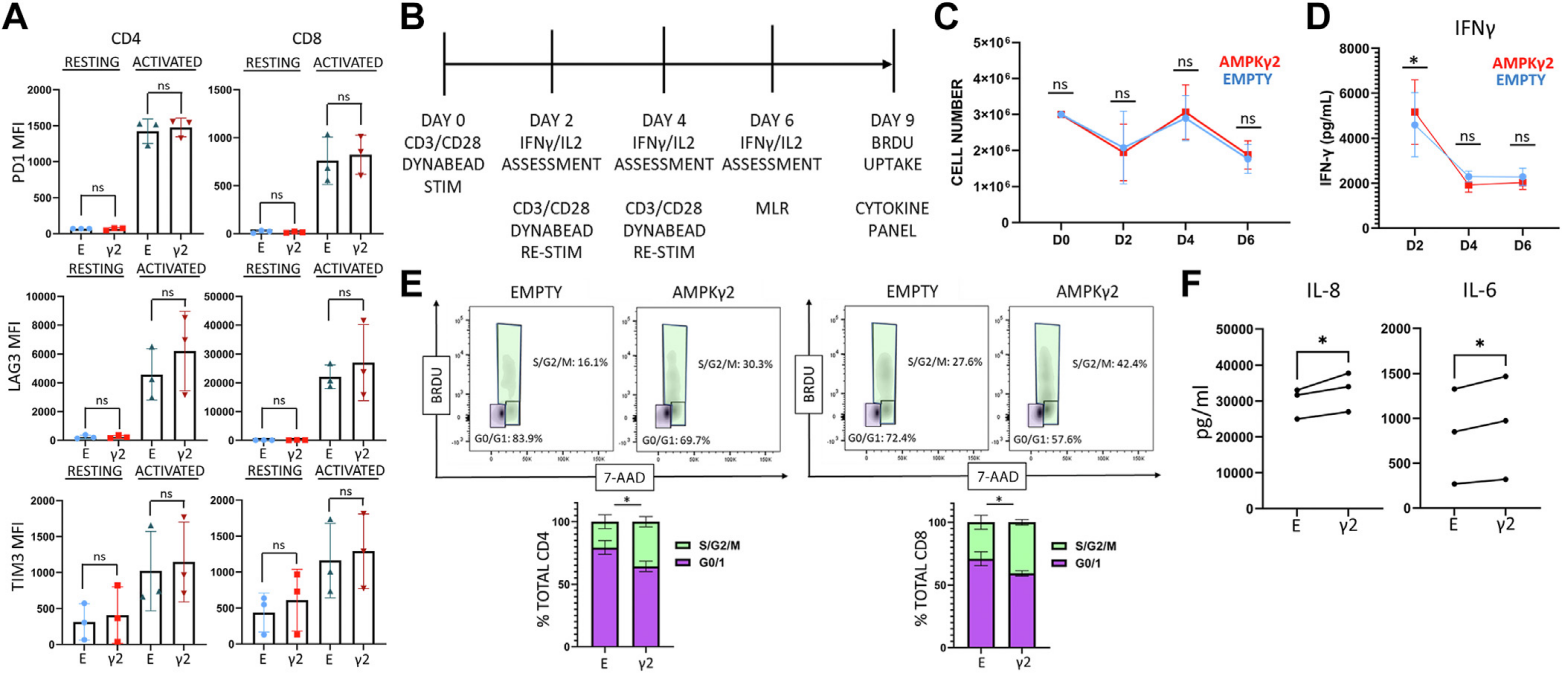
**AMPK transduction does not accelerate exhaustion.***A*, AMPKγ2- and empty-transduced cells were expanded *in vitro* in the presence of IL-2 and assessed on day 9 for cell surface expression of PD-1, Tim-3, and Lag-3 (resting) or restimulated with CD3/CD28 Dynabeads for 24 h and reassessed for PD-1, Tim-3, and Lag-3 expression (activated). *B*–*D*, a timeline of the *in vitro* exhaustion protocol (*B*), with cell counts at each timepoint (*C*) and IFNγ production measured by ELISA (*D*). *E* and *F*, after three consecutive restimulations, T cells were placed with allogeneic APCs for 72 h, incubated with BrdU for two additional hours, counterstained with 7-AAD, and measured for cell cycle analysis by flow cytometry (*E*). Media was also harvested from MLR cultures at 72 h and assessed for differences in cytokine expression between AMPK- and empty-transduced T cell cultures using LegendPlex analysis (*F*). Data represent three human donors. ∗*p* < 0.05 by paired Student’s *t* test. 7-AAD, 7-amino actinomycin D; AMPK, AMP-activated protein kinase; APC, antigen-presenting cell; IFNγ, interferon gamma; MLR, mixed leukocyte reaction.

Given the increased proliferation, enhanced glycolysis, and prolonged activation of AMPKγ2-transduced cells, we worried about potential exhaustion. Reassuringly, there was no significant difference in the expression of exhaustion markers PD1, Tim3, or LAG3 in AMPKγ2-transduced on day 9, nor did AMPK-transduced cells demonstrate an inability to upregulate activation markers upon restimulation, supporting the idea that AMPK-transduced cells were neither exhausted nor functionally impaired (Fig. 4*A*). We next assessed the function of AMPKγ2- *versus* empty-transduced cells, following an *in vitro* exhaustion protocol (44, 45). In this setting, expanded cells were restimulated with CD3/CD28 Dynabeads every 48 h over 6 days, with cell counts, IL-2, and interferon gamma (IFNγ) measured at each timepoint (Fig. 4*B*). After three serial stimulations, cells were placed into a mixed leukocyte reaction (MLR) against allogeneic antigen-presenting cells, harvested after 72 h, and cells examined for cell cycling by BRDU uptake and supernatant cytokine production as measured by LegendPlex analysis. During CD3/CD28 restimulation, AMPK-transduced cells showed no deficiency in cell counts (Fig. 4*C*) or cytokine production, initially making more IFNγ (Fig. 4*D*) and equivalent levels of IL-2 (Fig. S2). In the MLR, AMPK-transduced cells enhanced their proliferative capacity and mediated increased release of inflammatory cytokines IL-8 and IL-6 into the media (Fig. 4, *E* and *F*). Thus, despite increased proliferation and enhanced glycolytic activity, AMPK-activated cells were not more prone to exhaustion, instead demonstrating increased inflammatory capacity following repeat stimulation.

### AMPKγ2 overexpression enhances memory T cell yield

**Figure 5:**
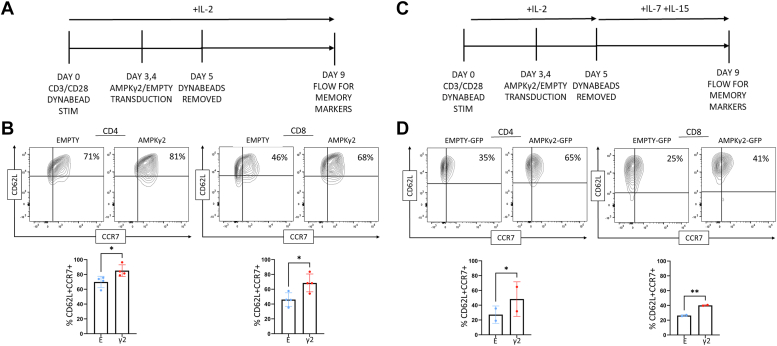
**AMPKγ2 transduction enhances yield of CD4 and CD8 memory T cells.***A* and *B*, AMPKγ2- and empty-transduced cells were expanded *in vitro* in the presence of IL-2 and assessed on day 9 for cell surface expression of CD62L and CCR7 to identify central memory-like T cells. *C* and *D*, a second group of cells were stimulated in IL-2 through day 5, and then expanded in IL-7 and IL-15 until day 9, when they were stained for CD62L and CCR7 expression. Bar graphs for *B* and *D* represent data from 2 to 4 individual human donors. ∗*p* < 0.05 and ∗∗*p* < 0.01 by paired Student’s *t* test. AMPK, AMP-activated protein kinase; CCR7, chemokine receptor 7; IL, interleukin.

We next sought to characterize whether changes driven by AMPKγ2 transduction impacted T cell differentiation. This feature was of particular interest given that adoptively transferred T cells with a memory-like phenotype function more effectively *in vivo* (13, 14, 46, 47). AMPK transduction increased the percentage of central memory-like T cells on day 9 of culture with increased coexpression of CD62L/CCR (chemokine receptor)7 (Fig. 5, *A* and *B*). Further, to assess whether this AMPK effect was additive in the presence of “memory-inducing” cytokines, we transduced T cells in IL-2, then cultured them in IL-7 and IL-15, a process shown to promote central memory cells (14, 48, 49). AMPK transduction again increased the yield of central memory-like T cells on day 9 of culture (Fig. 5, *C* and *D*).

### Increased AMPK activity improves T cell function during glucose restriction

**Figure 6:**
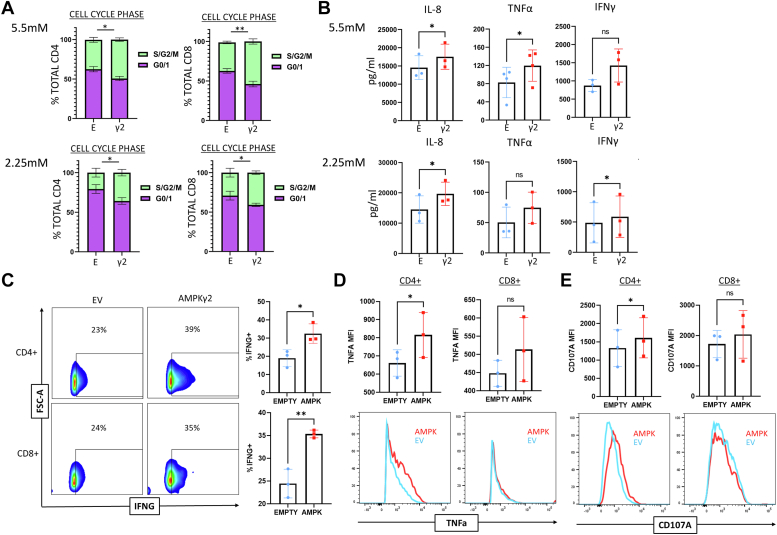
**AMPKγ2-transduced cells exhibit improved function during nutrient restriction.***A* and *B*, AMPK- and empty-transduced T cells were cultured for up to 12 days and then restimulated in 5.5 mM (physiologic) or 2.25 mM (*low*) glucose for 72 h. To measure cell cycling, cells were restimulated with TRANSACT for 72 h, cultured with BrdU for an additional 2 h, counter-stained with 7-amino actinomycin D, and assessed for cell cycling (*A*). To measure cytokine production, cells were plated 1:1 with allogeneic APCs and media harvested at 24 h for LegendPlex cytokine analysis (*B*). *C*–*E*, cells were restimulated overnight in the presence of brefeldin for 16 h, followed by staining for IFNγ (*C*), TNF (*D*), or CD107a (*E*). Representative flow plots are shown. Bar graphs represent data from 3 to 4 independent donors. ∗*p* < 0.05 and ∗∗*p* < 0.01 by paired Student’s *t* test. AMPK, AMP-activated protein kinase; APC, antigen-presenting cell; IFNγ, interferon gamma; TNF, tumor necrosis factor.

We then asked whether the metabolic changes enforced by AMPK modified T cell function. To start, we assessed proliferation (*via* BrdU incorporation) after 72 h of stimulation with T cell TransACT (Miltenyi). Interestingly, while there was no significant proliferative difference between groups in complete media (11 mM glucose) (Fig. S3*A*), there was a significant increase in cell cycling in AMPK-transduced cells under increasing glucose restriction (5 mM and 2.5 mM) (Fig. 6*A*). To evaluate inflammatory capacity, AMPK- and empty vector (EV)-transduced cells were activated *via* MLR for 72 h, media harvested, and cytokines measured by LegendPlex analysis. Again, cytokine production was similar in the 11 mM glucose cultures (Fig. S3*B*), but significantly increased for IL-8, IFNγ, and tumor necrosis factor (TNF) when glucose was restricted (Fig. 6*B*). In addition, when cytokine production was compared between 11 mM and 5.5 mM groups, AMPK-transduced cells either demonstrated increased cytokine production when glucose was restricted (Fig. S3*C*) or maintained cytokine production in situations where levels from EV-transduced cells diminished (Fig. S3*D*).

Importantly, cell counts did not differ between AMPKγ2- and EV-transduced cells after 72 h of MLR culturing, suggesting that cytokine production increased on a per cell basis (Fig. S3*E*). To corroborate these findings, AMPKγ2- and EV-transduced T cells were activated overnight with TransACT in the presence of brefeldin A, followed by intracellular cytokine analysis. AMPKγ2 overexpression increased the percentage of IFNγ+ cells in both CD4+ and CD8+ populations (Fig. 6*C*). While TNF+ percentages did not differ between groups, the TNF median fluorescence intensity was significantly upregaulted in CD4+ cells, suggesting higher total TNF production per cell (Fig. 6*D*). Finally, AMPKγ2-transduced CD4+ T cells also stained more brightly for the degranulation marker CD107a, with a trend towards increased CD107a staining in the CD8+ population (Fig. 6*E*). Together, these data highlight the increased capacity of AMPK-transduced cells to maintain or increase in function, in particular their inflammatory capacity, under conditions of glucose restriction.

### Increased AMPK activity differentially impacts CD4+ T cell subsets

**Figure 7:**
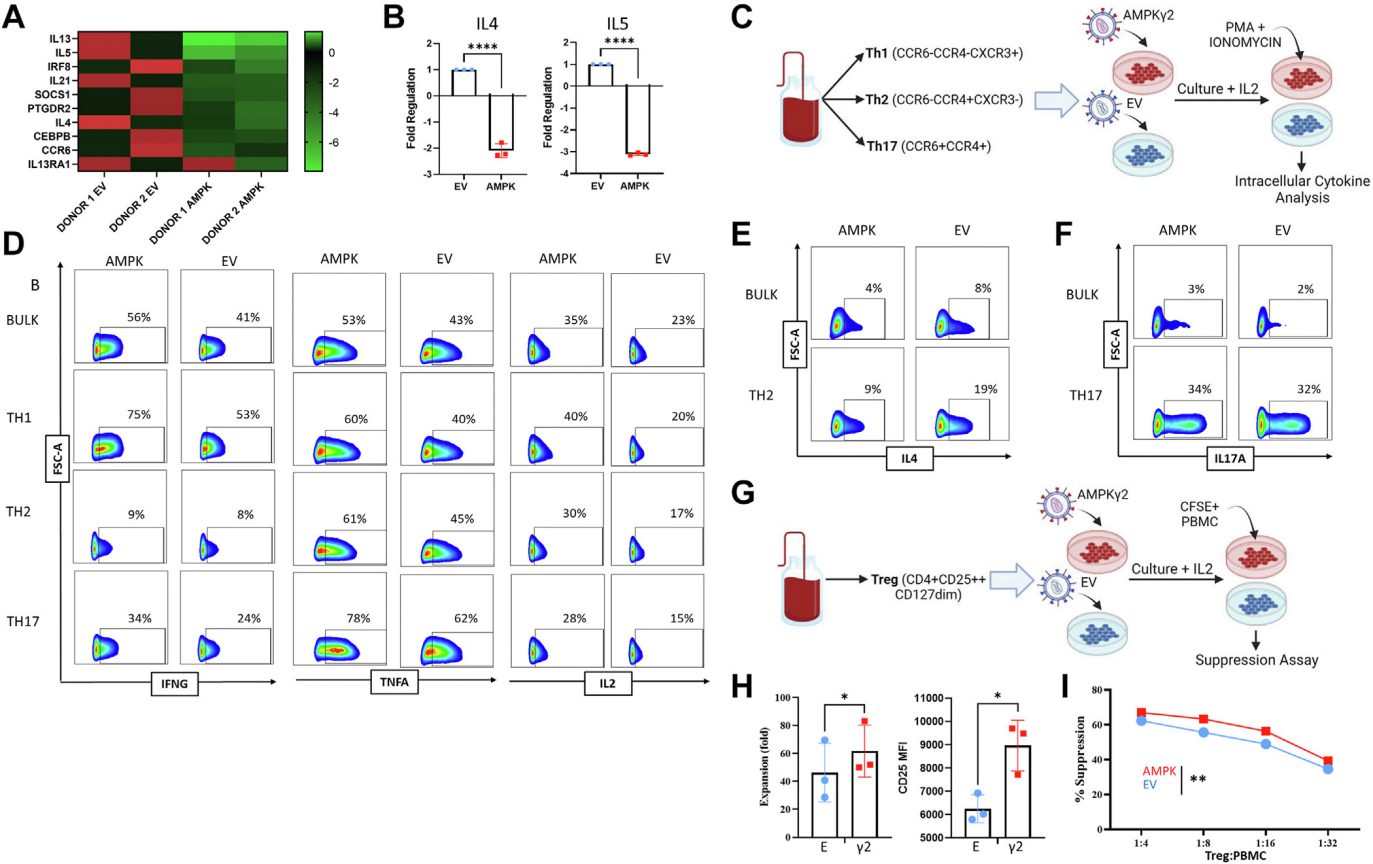
**Increased AMPK activity differentially impacts different CD4+ T cell subsets.** AMPKγ2- and empty-transduced CD4+ T cells were flow sorted and expanded to day 12, followed by RNA isolation and expression analysis using the Qiagen RT2 profiler array. Top ten differentially expressed genes between AMPKγ2- and empty-transduced CD4+ T cells from two independent donors are shown (*A*). Profiler results were independently verified for IL-4 and IL-5 expression using qRT-PCR and RNA from three human donors (*B*). *C*–*F*, fresh human CD4+ T cells were flow sorted into Th1, Th2, and Th17 populations based on chemokine receptor expression, separately transduced with either AMPKγ2 or EV controls, and expanded in IL-2 until day 10. Cells were then treated for 4 h with PMA and ionomycin, with monensin added for the final 3 h of incubation (*C*). Th1 cytokines IFNγ, TNF, and IL-2 were measured in each Th subset as well as bulk CD4+ T cells (*D*), while IL-4 was measured in Th2 as well as bulk CD4+ T cells (*E*), and IL17A was measured in Th17 and bulk CD4+ T cells (*F*). *G*–*I*, human Treg cells were flow sorted, transduced with AMPKg2 or EV control, and expanded in IL-2 (*G*). Expansion was calculated between days 3 and 5 of culture (*left*), with CD25 MFI measured by flow cytometry on day 5 (*H*). Treg suppression was measured by coculture with anti-CD3 stimulated CFSE+ PBMC at noted Treg:PBMC ratios on day 7, with suppression calculated 4 days later using the division index (*I*). ∗*p* < 0.05, ∗∗*p* < 0.01, and ∗∗∗∗*p* < 0.0001 by paired Student’s *t* test. AMPK, AMP-activated protein kinase; CFSE, carboxyfluorescein succinimidyl ester; EV, empty vector; IFNγ, interferon gamma; MFI, median fluorescence intensity; PBMC, peripheral blood mononuclear cell; TNF, tumor necrosis factor.

To gain a more comprehensive characterization of gene expression in CD4+ T helper subsets that might be impacted by AMPKγ2 overexpression, we pursued RT2 profiler analysis using the Human T Helper Cell Differentiation Array from Qiagen. RNA was recovered from day 9 bulk CD4+ AMPKγ2-and EV-transduced T cells, converted to cDNA, and evaluated *via* profiler array. Analysis of the ten most downregulated genes across two individual donors demonstrated a striking decrease in expression of T helper type 2 (Th2) genes, including IL-4, IL-5, and IL-13 (Fig. 7*A*). This drop in cytokine expression was independently confirmed by quantitative RT-PCR, where transcripts for both IL-4 and IL-5 were reduced in AMPKγ2-transduced cells (Fig. 7*B*).

Overexpression of AMPKγ2 in bulk T cells would most closely reflect augmenting cellular therapies in the clinic. However, we also wished to determine the impact of AMPKγ2 overexpression on lineage committed T cell subsets. To this end, human T cells were flow sorted into traditional Th1/Th2/Th17 subsets based upon a combination of CCR4, CCR6, and CXC chemokine receptor 3 expression (Fig. 7*C*) as described by others (50). Each individual subset was then stimulated with Dynabeads, transduced with AMPKγ2 or an EV, and expanded in IL-2 until day 10, followed by evaluation of intracellular cytokine production. Once again, bulk CD4+ T cells increased production of Th1 cytokines IFNγ, TNF, and IL2, an effect similarly enhanced in Th1 and Th17 subsets and for TNF and IL-2 in Th2 cells (Fig. 7*D*). Consistent with the array data, IL-4 protein expression decreased in both bulk CD4s and the Th2 subgroup, with half as many T cells producing IL-4 in AMPKγ2 compared to EV control cells (Fig. 7*E*). Meanwhile, bulk CD4 T cells had minimal IL-17 expression, while Th17 cells had elevated but equivalent levels of this lineage-defining cytokine between AMPKγ2- and EV-transduced groups (Fig. 7*F*).

To investigate whether AMPKγ2 overexpression differentially impacted Tregs, naïve human thymic Treg (tTreg) were purified from peripheral blood mononuclear cells (PBMCs) by first column sorting on CD25+ cells, followed by flow-sorting for CD4+, CD8−, CD25 high, CD127−, CD45RA+ cells. Purified tTreg were then stimulated with a K562 cell line engineered to express CD86 and the high-affinity Fc receptor (CD64) (37) and cultured with recombinant IL-2 (Fig. 7, G). Expanded tTreg cells were transduced with AMPKγ2 by lentiviral transduction and assessed for proliferation/expansion, FoxP3 expression, CD25 positivity, and suppressive capacity. FoxP3 levels were maintained in all cultured tTreg independent of AMPKγ2 transduction (Fig. S4). In contrast, both expansion and CD25 expression increased in Treg transduced with AMPKγ2 (Fig. 7*H*), similar to AMPKγ2 transduction of bulk T cells. Finally, Treg suppressive capacity was assessed by plating tTreg ± AMPKγ2 with varying concentrations of conventional T cells (Tcon) labeled with carboxyfluorescein succinimidyl ester (CFSE). Regulatory cells overexpressing AMPKγ2 had increased suppressive capacity across a range of Treg/Tcon ratios (Fig. 7*I*). Together, these data demonstrate that AMPKγ2 transduction, with its attendant increase in AMPK signaling, diminishes Th2 phenotypes, directs a more inflammatory response in bulk as well as Th1 and Th17 T cells, and promotes a more suppressive phenotype when transduced specifically into Treg.

## Discussion

Adoptive cellular therapies, including CAR-T cells, are often limited by terminal differentiation of the cellular product and metabolic exhaustion of the transferred cells. These less desirable phenotypes result from a number of secondary causes including *in vitro* expansion protocols driving metabolic exhaustion, the immunosuppressive and nutrient-deplete *in vivo* tumor environment, and the exhausted status of patient-derived cells. Methods to ameliorate this metabolic and functional insufficiency are needed to improve and expand the use of adoptive cellular therapies. One unique advantage of *in vitro* expansion is the opportunity to alter T cell phenotypes and thereby improve their subsequent function upon return to patients. Manipulating a variety of metabolic pathways has been attempted, but these alterations have seen limited clinical application to date; due, in part, to the restricted expansion that comes as a byproduct of the methods used to promote oxidative metabolism (12).

In our model, lentiviral transduction of the regulatory AMPKγ2 subunit increased AMPK activity, a major regulator of cellular metabolism and guardian of mitochondrial function. Our data suggest that increased AMPK signaling through AMPKγ2-containing heterotrimers enhances both oxidative metabolism and glycolysis, allowing increased *in vitro* expansion of transduced cells without driving exhaustion. Importantly, the enhanced respiration seen in AMPKγ2-transduced cells increased further following TCR activation. This powerful finding suggests that our method of metabolically reprogramming the AMPK pathway is likely to provide benefit when underperforming cells need it most (*i.e.*, during T cell stimulation). We further found that this method of activating AMPK increased the yield of central memory T cells, even in the presence of “memory-inducing” cytokines IL-7 and IL-15, consistent with a more active role for AMPK in memory *versus* effector T cells (51). This finding is of particular interest given recent data in leukemia patients undergoing CART therapy, which suggests a correlation between long-term cures and the pool of CD4+ memory T cells (52).

Bulk AMPKγ2-transduced T cells outperformed control cells in both proliferation and proinflammatory cytokine production when activated under physiologic and low glucose conditions, suggesting that transduced cells may have an advantage in glucose-restricted settings *in vivo* (53). The fact that these changes were not seen in 11 mM high glucose media resonates with other studies in which AMPK specifically rescues cells only in the setting of cellular stress (54). In evaluating the mechanism underlying these functional improvements, we found increases in proteins related to both mitochondrial biogenesis and fusion, consistent with known effects of AMPK signaling (1, 16). This combination of mitochondrially-centered changes also likely contributes to the notable increase in SRC, which has been a hallmark of T cells capable of improved functional ability *in vivo* (1, 7). In addition, upregulation of ULK1 and its effect on mitophagy may further contribute to overall mitochondrial health and improved oxidative efficiency (16, 55), while the notable increase in GLUT1 expression explains improved functionality under low glucose conditions.

When the functionality of different CD4+ subsets was probed following AMPKγ2 transduction, we were intrigued to find that AMPK amplified inflammatory function in conventional Th subsets but also increased suppressive function in Treg. These findings suggest that AMPK may not independently reprogram CD4 T cell function (56), but rather provides the metabolic capacity to reinforce existing programming (inflammation in Tcon, suppression in Treg). It is notable that increased AMPK signaling specifically counteracts Th2 specific programming, both in bulk CD4s and within the purified Th2 subset. This finding is supported by reports in the literature where AMPK specifically downregulates Th2 programming through its impact on Mtorc2 (57). However, a portion of this influence may also be secondary to the culture conditions used in these experiments, which naturally promote a Th1 over Th2 expansion and phenotype. Future studies examining the impact of AMPKγ2 expression specifically on Th2 differentiation, using cells grown in Th2 culture conditions, may better delineate AMPK’s role in this process.

In addition to the expected changes in oxidative metabolism, AMPK-overexpressing T cells also enhanced extracellular acidification, suggesting increased glycolysis. This effect is consistent with previous reports on AMPK signaling in which increased glycolysis occurred alongside increased expression of GLUT transporters (58) and enhanced phosphorylation of 6-phosphofructo-2-kinase (21, 39). Increased flux through the proximal steps of glycolysis could also feed an increase in oxidative metabolism by shuttling more pyruvate to the tricarboxylic acid cycle (53). Elevated glycolysis aligns with the increased growth and proliferation of AMPKγ2-transduced cells, where more cells are found within active phases of the cell cycle. At least a portion of this increased proliferation may be secondary to a heightened ability of cells to respond to IL2, as suggested by our data on increased CD25 expression. Reassuringly, other models have noted a similar link between AMPK and upregulation of CD25-mediated IL2 signaling (59). Notably, despite increased *in vitro* expansion, there was no evidence of impending exhaustion in AMPKγ2-transduced cells as measured by PD1, TIM3, and LAG3 expression and no impairment in upregulation of these markers upon further TCR stimulation. Further, AMPK-transduced cells outperformed controls following a program of *in vitro* exhaustion, supporting the view that AMPKγ2-transduced cells are capable of full activation despite prolonged activity *in vitro*.

Increased cell proliferation also correlated with an increase in mTOR activity, a finding contradictory to the traditionally accepted antagonism between AMPK and mTOR signaling (18, 60). Two potential hypotheses may explain these findings: First, it is possible that persistently increased AMPK signaling creates a more metabolically efficient cell through promotion of mitochondrial health. Because of this ongoing efficiency, AMPKγ2-transduced cells maintain a higher level of proliferation as they expand in culture, which requires a subsequent compensatory increase in mTOR signaling. Another possible hypothesis involves the role of AMPKγ2 *versus* AMPKγ1-containing heterotrimers. Other systems have demonstrated differences in activation and pathway targeting dependent upon AMPKγ isoform usage (61, 62). In the present case, AMPKγ2-heterotrimers may simply be less involved with mTOR regulation, allowing mTOR signaling to increase as AMPKγ2-signaling simultaneously creates more metabolically efficient cells. Indeed, ours is not the only model where AMPK and mTOR activity are concurrently upregulated (18, 63, 64), but additional work will be required to understand signaling differences enabled by γ1 *versus* γ2 regulatory isoforms. What is clear from the present study is that AMPKγ2 overexpression does not impact AMPKγ1 *expression* in human T cells. However, whether the role of AMPKγ1 is impacted by AMPKγ2 overexpression remains the focus for further investigations.

Another advantage of using AMPKγ2 overexpression to increase AMPK signaling involves the ability to augment AMPK without enacting cellular starvation signals. Given that AMPK is also activated downstream of the TCR, where it tailors T cell responses to energy availability (21), we hypothesize that AMPK activation in replete nutrients engages different downstream targets than cells experiencing nutrient starvation. Because AMPKγ2 transduction does not rely on cellular starvation, but likely augments endogenous TCR-linked AMPK signaling, this approach may allow for deeper understanding of the connection between AMPK and TCR-driven activation and growth. Indeed, such a connection may help explain why memory T cells, which have increased AMPK activity, also show enhanced proliferation and cytokine generation upon reactivation (65).

Based on a wealth of data from the literature, T cells with increased oxidative metabolism and limited *in vitro* differentiation are expected to persist longer and perform better *in vivo* (10, 12, 14, 15). Thus, the next step will be to trial transduction of AMPKγ2 in T cells in a variety of animal models to assess the positive effects of enhanced AMPK activity on *in vivo* cellular functions. Indeed, it could be envisioned that multiple cell-based therapies, including the metabolic reprogramming of tumor-infiltrating lymphocytes or the reshaping of an antiviral immune response, may benefit from improved T cell expansion *in vitro* and potently enhanced inflammatory functions in nutrient-deplete microenvironments *in vivo*. In addition, AMPK’s ability to augment Treg suppressive capacity, enhance CD25 expression, and promote Treg expansion, could improve cellular therapies where the goal is to downregulate inflammation, including in cases of autoimmunity or solid organ transplantation. Fortuitously, many adoptive cellular therapies already require lentiviral or retroviral transduction, coupling nicely with our genetic manipulation of AMPK to potentially enhance T cell efficacy *in vivo* long after transfer.

In summary, increasing AMPK activity in primary human T cells, through lentiviral-driven overexpression of AMPKγ2, upregulates oxidative metabolism, decreases cellular differentiation, and improves *in vitro* expansion. Further, the resulting T cells demonstrate a decreased likelihood of developing into Th2 cells, with promotion of an inflammatory phenotype in bulk and conventional Th subsets and increased suppressive capacity when activity is driven specifically in Treg. Together, these characteristics provide an attractive method to improve the function of current adoptive cellular therapies and work is ongoing to assess the potential contribution of this modification to multiple T cell–based approaches *in vivo*.

## Experimental procedures

### Virus production

The AMPKγ2 sequence was amplified from a commercially available plasmid (Addgene #23689) and cloned into either a pSICO (Addgene #31847) or pHR (similar to Addgene #14858, kind gift from Jason Lohmueller, UPMC Hillman Cancer Center) backbone, followed by addition of a T2A linker and either GFP, tagBFP, or RQR8 tag (37). Transformed bacterial cultures were grown overnight in Terrific Broth (Sigma-Aldrich) and plasmids isolated using QIAGEN QIAmp Miniprep Plasmid Isolation Kit 250. HEK293Ts (American Type Culture Collection) were cultured in Dulbecco's modified Eagle's medium (Gibco #11966-025) containing 10% fetal bovine serum (FBS), Pen Strep, 2 mM L-Glutamine, and nonessential amino acids. Early passage cells were transfected using an Invitrogen Lipofectamine 3000 Transfection kit with 2500 ng of RSV-REV, PMD-2G, and PRRE plasmid and 10,000 ng of either AMPKγ2-t2a-GFP/RQR8 or t2a-GFP/RQR8 “empty” plasmid. After 24 h, supernatant was replaced with Iscove's Modified Dulbecco's Medium (Gibco #12440-053) containing 10% FBS. Supernatant containing viral particles was harvested at 48 and 72 h, combined with Lenti-Pac (GeneCopoeia), and incubated at 4 °C overnight. Viral supernatants were then centrifuged at 3500*g* for 25 min at 4 °C, resuspended in Dulbecco's modified Eagle's medium, and either frozen at −80 °C or used immediately.

### T cell isolation, transduction, and culture

De-identified buffy coats were obtained from healthy human donors (Vitalant), diluted with PBS, layered over lymphocyte separation medium (MPbio), and centrifuged at 400×*g* and 25° for 20 min with no brake. The PBMC layer was removed and T cells isolated using the Miltenyi Biotec Human Pan T cell isolation kit. Purified T cells were resuspended in AIM-V +5% serum replacement (SR; Gibco #A25961-01) and plated with Dynabeads human T-activator CD3/CD28 for T cell expansion and activation (Thermo Fisher Scientific) at a 2:1 ratio for 48 h. Transduction was then performed per manufacturer’s instructions utilizing retronectin-coated plates (Takara). Cells were removed from Dynabeads by magnetic separation on day 5 poststimulation and expanded in AIM-V media with 5% SR containing IL-2 at 100 IU/ml, or with IL-7 and IL15 at 20 ng/ml. Further assessments were performed between day 9 and 12 of culture. For restimulation experiments, cells were replated with Dynabeads at a 1:1 ratio for up to 72 h. Transduced T cells used in bulk assays (such as the Seahorse), were flow sorted on a BD FACs Aria to enrich for the transduced target population prior to assay analysis.

### Treg purification, culture, and transduction

Naïve human tTreg were purified, expanded, and banked as described previously (66). Briefly, naïve human PB tTreg (CD4+25 + 127–45RA+) were sort-purified from PBMCs (Ficoll-Hypaque, Amersham Biosciences) in a two-step procedure in which CD25+ cells were first enriched from PBMCs by AutoMACS (PosselD2) with GMP grade anti-CD25 microbeads (Miltenyi Biotec). CD25 high cells were stained with CD4, CD8, CD25, CD127, and CD45RA and sorted *via* FACSAria as CD4+, CD8−, CD25 high, CD127−, and CD45RA+. Purified naïve tTreg were incubated with anti-CD3 mAb (Miltenyi Biotec) and further stimulated (1:1) with a K562 cell line engineered to express CD86 and the high-affinity Fc receptor (CD64) and which had been irradiated with 10,000 cGy. Naive tTreg were cultured in X-Vivo-15 media (BioWhittaker) supplemented with 10% human AB serum (Valley Biomedical). Recombinant IL-2 (300 IU/ml, Chiron) was added on day 2 and maintained for culture duration. Cultures were maintained at 0.25 to 0.5× 10e6 viable nucleated cells/ml, being split every 2 to 3 days as needed. Aliquots were frozen down on day 14. When needed, aliquots of expanded tTreg where thawed, incubated overnight in complete media + IL-2, and transduced the following day with high-titer AMPKγ2 lentivirus. On day 2, transduced tTreg were restimulated with KT64/86 and expanded an additional 7 days. Flow phenotyping was performed 5 days after restimulation to assess transduction efficiency and again on d7.

The *in vitro* suppressive capacity of expanded tTregs was assessed on day 7 using a CFSE inhibition assay as previously published (66). Briefly, PBMCs were labeled with CFSE (Invitrogen) and stimulated with anti-CD3 mAb-coated beads (Dynal) ± cultured tTreg (ratios of tTregs/PBMNCs, 1:2–1:32). On day 4, cells were stained with antibodies to CD4 and CD8 and suppression was determined by measuring the Division Index (FlowJo, https://www.flowjo.com, TreeStar) ± tTregs. To note, tTregs suppressed CD4 and CD8 T cell responses equivalently, with representative CD4 data presented.

### Protein isolation and immunoblot

CD8+ and CD4+ T cells were first separated on day 9 utilizing the STEMCELL CD8 positive selection kit according to manufacturer’s instructions (Stemcell Technologies) and plated overnight in AIM-V + 5% SR. The following day, GFP+ cells were flow sorted directly into 10% trichloroacetic acid and lysates centrifuged at 16000*g* at 4 °C for 10 min, washed twice in ice cold acetone, resuspended in solubilization buffer (9 M urea containing 1% DTT and 2% Triton X and NuPAGE lithium dodecyl sulfate sample buffer 4× (Invitrogen) at a 3:1 ratio), and heated at 70 °C for 10 min (67). Protein gel electrophoresis was performed on ice using NuPAGE 4 to 12% Bis-Tris Protein Gels (Invitrogen) at 135 V. In some cases, protein samples were heated to 95 °C for 5 min prior to gel loading. Protein was transferred to Invitrolon 0.45 μm polyvinylidene difluoride membranes (Invitrogen) at 30 V on ice for 1 h. Membranes were blocked in Tris-buffered saline–Triton containing 5% nonfat milk and immunoblotting performed according to the Cell Signaling Technologies Western Blot Protocol. Blots were stripped for 10 min (Restore PLUS Western Blot Stripping Buffer, Thermo Fisher Scientific) prior to reprobing. Antibodies used for immunoblotting are listed in Table S1. Blots were developed with Super Signal West Femto chemiluminescence reagents (Thermo Fisher Scientific), detected by CL-XPosure Film (Thermo Fisher Scientific), and scanned in grayscale with an Epson V600 scanner. Images were cropped using ImageJ Software (https://imagej.net/ij/download.html; version 1.47 T), inverted, and densitometry quantitated in an area encompassing the largest band, followed by quantitation of all subsequent bands using the same two-dimensional area.

### Flow cytometry

Cells were washed with PBS + 2% FBS before staining with antibodies at 1:100 dilution for 30 min. For intracellular stains, cells were fixed per manufacturer’s instructions using Fix/Perm kit (Cat #:88-8824-00, Invitrogen) and then stained with antibodies at 1:100 dilution. Antibodies and other flow cytometry reagents are listed in Table S2. MitoTracker Red (Invitrogen) staining was performed at 1 nM in PBS with incubation at 37° for 15 min. BrdU analysis was performed utilizing the Phase-Flow kit per manufacturer’s instructions (BioLegend), with cells cultured in BrdU for 2 h prior to staining. Flow data was captured on a BD Fortessa analyzer (BD Biosciences) and evaluated using FlowJo software (version 10.1, Tree Star). Cells were gated by forward and side scatter to identify lymphocyte population, and then gated on GFP+ or CD34+ (RQR8 marker) cells for downstream analysis.

### Seahorse Mito stress assay

The Seahorse XF Cell Mito Stress Test Kit (Agilent; Catalog #103015-100) was run on a Seahorse XFe96 Bioanalyzer (Agilent) to determine basal and maximal OCR, SRC, and ECAR for transduced T cells. T cells were plated in assay media (XF Base media (Agilent) with glucose (25 mM), sodium pyruvate (2 mM) and L-glutamine (4 mM) (Gibco), pH 7.4 at 37  °C) on a Seahorse cell culture plate coated with Cell-Tak (Corning) at 1 × 10^5^ cells/well. After adherence and equilibration, basal ECAR and OCR readings were taken for 30 min. Cells were then stimulated with oligomycin (2 μM), carbonyl cyanide 4-(trifluoromethoxy) phenylhydrazone (1 μM), and rotenone/antimycin A (0.5 μM) to obtain maximal respiratory and control values. Assay parameters were as follows: 3 min mix, no wait, 3 min measurement, repeated for three cycles at baseline and after each injection. SRC was calculated as the difference between basal and maximal OCR values obtained after trifluoromethoxy) phenylhydrazone uncoupling. The XF Mito Stress Test report generator and Agilent Seahorse analytics were used to calculate parameters from Wave software (https://www.waveapps.com; Agilent, Version 2.6.1.53).

### Cytokine multiplex and ELISA analysis

Supernatants were assessed for cytokine production using the LegendPlex Human Inflammation Panel 1 (13-plex) in a V-bottom plate per the manufacturer’s instructions (BioLegend). Flow cytometry data were acquired on a BD Fortessa analyzer (BD Biosciences) and assessed using FlowJo software (version 10.7, Tree Star). Where indicated, ELISA to detect human IL-8 was done using culture supernatants at 1:100 dilution in a Quantikine ELISA kit (R&D Systems) per manufacturer’s instructions. One sample run by LegendPlex did not have sufficient volume to be further assessed by ELISA.

### Mixed leukocyte reaction

On day 9 of culture, cells were flow sorted for GFP positivity, labeled with CellTrace Violet (Invitrogen), and placed at 3 × 10^5^/well in culture with 3 × 10^5^ allogeneic non-T cell antigen-presenting cells irradiated at 20 Gy. MLRs were performed in 96-well round-bottom plates in Roswell Park Memorial Institute media (RPMI) with varying levels of glucose. Physiologic glucose media was obtained by mixing no glucose RPMI (Gibco Cat #11879-020) 1:1 with standard RPMI media (Gibco Cat #11875-085) for a final glucose concentration of 1 g/L (5.5 mM). Low glucose RPMI was obtained by mixing no glucose RPMI 1:3 with standard RPMI media for a final glucose concentration of 0.5 g/L (2.25 mM). After 3 to 4 days, media was collected for cytokine analysis and cells were assessed by flow cytometry, with responding cells identified by decreased levels of CellTrace Violet. Cell counts were assessed by flow cytometry using CountBright beads (Thermo Fisher Scientific) as per manufacturer’s instructions.

### RT2 profiler array and RT-PCR

Cell lysates from 5e6 CD4 T cells on day 9 of culture were suspended in RLT buffer and processed through a QiaShredder (Qiagen) prior to being frozen at –80 °C. Total RNA was later isolated from homogenized lysates using the Qiagen RNeasy Plus Mini Kit according to manufacturer’s instructions. The RNA was quantified by Nanodrop, and 500 ng of total RNA was synthesized into cDNA using the Qiagen RT^2^ First Strand Kit following the recommended protocol.

Real-time PCR was then carried out using the Qiagen RT^2^ Profiler PCR Human T Helper Cell Differentiation Array (Format C, for use with the Applied Biosystems StepOnePlus real-time cycler) in combination with the corresponding Qiagen RT^2^ SYBR Green ROX qPCR Mastermix materials. For 96-wells, 1485ul of RT^2^ SYBR Green Mastermix, 1373 ul of nuclease-free water, and 102 ul of cDNA were combined. Twenty-five microliters was dispensed per well, changing the tips between rows to prevent cross contamination. The plate was run under standard qPCR default protocol settings (cycling stage 60C 1 min; Melting Curve stage: during ramp from 60C to 95C; 40 cycles).

For quantitative RT-PCR confirmation, 5e6 T cells were lysed in RLT buffer (Qiagen RNeasy Plus Mini Kit) and lysates homogenized using the Qiagen QIAshredders per manufacturer’s directions. RNA was isolated from homogenized lysates using the RNeasy Plus Mini Kit according to manufacturer’s recommended protocol and RNA quantitated by NanoDrop. One microgram of RNA was reverse transcribed to cDNA using the Bio-Rad iScript cDNA Reaction Kit (per kit’s instructions). Using Bio-Rad’s SYBR Green Master Mix reagent, 4 ul of cDNA was placed into 36 ul of water, master mix, and primers and quantitative PCR reactions run in triplicate of 10 ul each on an Applied Biosystem QuantStudio3 cycler with the standard protocol of 95C and 60C over 40× cycles. RT-PCR primer sequences are listed in Table S3.

### Statistics

Graphing and statistical analysis was performed using GraphPad Prism for Windows (version 9.0.1, www.graphpad.com). Paired two-tailed Student’s *t* test or one-way ANOVA were used to determine statistical significance. All samples with variability between data points were run through a two-sided Grubbs’ test for outliers using GraphPad Prism software. After Grubbs’ analysis, one monocyte chemoattractant protein-1 sample and one IFNγ sample, each from the 11 mM and 5.5 mM MLRs were eliminated as outliers (making three independent donors instead of four). Unless noted otherwise, data are displayed as mean ± SEM. In all cases ∗*p* < 0.05, ∗∗*p* < 0.01, ∗∗∗*p* < 0.001, and ∗∗∗∗*p* < 0.0001.

### Study approval

All studies on human cells were designated Exempt status by the University of Pittsburgh Institutional Review Board.

## Data availability

RT2 Profiler array data is available *via* the Gene Expression Omnibus online database at https://www.ncbi.nlm.nih.gov/geo/ under accession number GSE252375. All remaining data are contained within the manuscript.

## Supporting information

This article contains supporting information.

## Conflict of interest

Drs Byersdorfer and Braverman are listed as coinventors on a patent application currently under consideration covering the use of increased AMPK signaling in human T cells. The remaining authors declare no conflicts of interest with the contents of this article.

## References

[1] Geltink R.I.K., Kyle R.L., Pearce E.L. (2018). Unraveling the complex interplay between T cell metabolism and function. Annu. Rev. Immunol..

[2] Rivadeneira D.B., Delgoffe G.M. (2018). Antitumor T-cell reconditioning: improving metabolic fitness for optimal cancer immunotherapy. Clin. Cancer Res..

[3] Rangel Rivera G.O., Knochelmann H.M., Dwyer C.J., Smith A.S., Wyatt M.M., Rivera-Reyes A.M. (2021). Fundamentals of T cell metabolism and strategies to enhance cancer immunotherapy. Front. Immunol..

[4] Yu Y.-R., Imrichova H., Wang H., Chao T., Xiao Z., Gao M. (2020). Disturbed mitochondrial dynamics in CD8+ TILs reinforce T cell exhaustion. Nat. Immunol..

[5] Vardhana S.A., Hwee M.A., Berisa M., Wells D.K., Yost K.E., King B. (2020). Impaired mitochondrial oxidative phosphorylation limits the self-renewal of T cells exposed to persistent antigen. Nat. Immunol..

[6] Scharping N.E., Menk A.V., Moreci R.S., Whetstone R.D., Dadey R.E., Watkins S.C. (2016). The tumor microenvironment represses T cell mitochondrial biogenesis to drive intratumoral T cell metabolic insufficiency and dysfunction. Immunity.

[7] Menk A.V., Scharping N.E., Rivadeneira D.B., Calderon M.J., Watson M.J., Dunstane D. (2018). 4-1BB costimulation induces T cell mitochondrial function and biogenesis enabling cancer immunotherapeutic responses. J. Exp. Med..

[8] Drent E., Poels R., Ruiter R., van de Donk N.W.C.J., Zweegman S., Yuan H. (2019). Combined CD28 and 4-1BB costimulation potentiates affinity-tuned chimeric antigen receptor-engineered T cells. Clin. Cancer Res..

[9] Nabe S., Yamada T., Suzuki J., Toriyama K., Yasuoka T., Kuwahara M. (2018). Reinforce the antitumor activity of CD8+ T cells via glutamine restriction. Cancer Sci..

[10] Buck M.D., O’Sullivan D., Klein Geltink R.I., Curtis J.D., Chang C.-H., Sanin D.E. (2016). Mitochondrial dynamics controls T cell fate through metabolic programming. Cell.

[11] Dumauthioz N., Tschumi B., Wenes M., Marti B., Wang H., Franco F. (2021). Enforced PGC-1α expression promotes CD8 T cell fitness, memory formation and antitumor immunity. Cell Mol. Immunol..

[12] Sukumar M., Liu J., Ji Y., Subramanian M., Crompton J.G., Yu Z. (2013). Inhibiting glycolytic metabolism enhances CD8+ T cell memory and antitumor function. J. Clin. Invest..

[13] Pilipow K., Scamardella E., Lugli E. (2020). Generating stem-like memory T cells with antioxidants for adoptive cell transfer immunotherapy of cancer. Meth. Enzymol..

[14] Klebanoff C.A., Finkelstein S.E., Surman D.R., Lichtman M.K., Gattinoni L., Theoret M.R. (2004). IL-15 enhances the *in vivo* antitumor activity of tumor-reactive CD8+ T cells. Proc. Natl. Acad. Sci. U. S. A..

[15] Hurst K.E., Lawrence K.A., Robino R.A., Ball L.E., Chung D., Thaxton J.E. (2020). Remodeling translation primes CD8+ T-cell antitumor immunity. Cancer Immunol. Res..

[16] Kang S.W.S., Haydar G., Taniane C., Farrell G., Arias I.M., Lippincott-Schwartz J. (2016). AMPK activation prevents and reverses drug-induced mitochondrial and hepatocyte injury by promoting mitochondrial fusion and function. PLoS One.

[17] Hardie D.G., Ross F.A., Hawley S.A. (2012). AMPK: a nutrient and energy sensor that maintains energy homeostasis. Nat. Rev. Mol. Cell Biol..

[18] Kazyken D., Magnuson B., Bodur C., Acosta-Jaquez H.A., Zhang D., Tong X. (2019). AMPK directly activates mTORC2 to promote cell survival during acute energetic stress. Sci. Signal..

[19] Rabinovitch R.C., Samborska B., Faubert B., Ma E.H., Gravel S.-P., Andrzejewski S. (2017). AMPK maintains cellular metabolic homeostasis through regulation of mitochondrial reactive oxygen species. Cell Rep..

[20] Herzig S., Shaw R.J. (2018). AMPK: guardian of metabolism and mitochondrial homeostasis. Nat. Rev. Mol. Cell Biol..

[21] Blagih J., Krawczyk C.M., Jones R.G. (2012). LKB1 and AMPK: central regulators of lymphocyte metabolism and function. Immunol. Rev..

[22] Zimmermann K., Baldinger J., Mayerhofer B., Atanasov A.G., Dirsch V.M., Heiss E.H. (2015). Activated AMPK boosts the Nrf2/HO-1 signaling axis--A role for the unfolded protein response. Free Radic. Biol. Med..

[23] Li Q., Wang Y., Wu S., Zhou Z., Ding X., Shi R. (2019). CircACC1 regulates assembly and activation of AMPK complex under metabolic stress. Cell Metab..

[24] Salminen A., Kaarniranta K. (2012). AMP-activated protein kinase (AMPK) controls the aging process via an integrated signaling network. Ageing Res. Rev..

[25] Ren Y., Shen H.-M. (2019). Critical role of AMPK in redox regulation under glucose starvation. Redox Biol..

[26] Rao E., Zhang Y., Zhu G., Hao J., Persson X.-M.T., Egilmez N.K. (2015). Deficiency of AMPK in CD8+ T cells suppresses their anti-tumor function by inducing protein phosphatase-mediated cell death. Oncotarget.

[27] Chamoto K., Chowdhury P.S., Kumar A., Sonomura K., Matsuda F., Fagarasan S. (2017). Mitochondrial activation chemicals synergize with surface receptor PD-1 blockade for T cell-dependent antitumor activity. Proc. Natl. Acad. Sci. U. S. A..

[28] Ross F.A., MacKintosh C., Hardie D.G. (2016). AMP-activated protein kinase: a cellular energy sensor that comes in 12 flavours. FEBS J..

[29] Tamás P., Hawley S.A., Clarke R.G., Mustard K.J., Green K., Hardie D.G. (2006). Regulation of the energy sensor AMP-activated protein kinase by antigen receptor and Ca2+ in T lymphocytes. J. Exp. Med..

[30] Jäger S., Handschin C., St-Pierre J., Spiegelman B.M. (2007). AMP-activated protein kinase (AMPK) action in skeletal muscle *via* direct phosphorylation of PGC-1alpha. Proc. Natl. Acad. Sci. U. S. A..

[31] Cantó C., Auwerx J. (2009). PGC-1alpha, SIRT1 and AMPK, an energy sensing network that controls energy expenditure. Curr. Opin. Lipidol..

[32] Tamargo-Gómez I., Mariño G. (2018). AMPK: regulation of metabolic dynamics in the context of autophagy. Int. J. Mol. Sci..

[33] Joseph B.K., Liu H.-Y., Francisco J., Pandya D., Donigan M., Gallo-Ebert C. (2015). Inhibition of AMP kinase by the protein phosphatase 2A heterotrimer, pp2appp2r2d. J. Biol. Chem..

[34] Sanders M.J., Grondin P.O., Hegarty B.D., Snowden M.A., Carling D. (2007). Investigating the mechanism for AMP activation of the AMP-activated protein kinase cascade. Biochem. J..

[35] Ross F.A., Jensen T.E., Hardie D.G. (2016). Differential regulation by AMP and ADP of AMPK complexes containing different γ subunit isoforms. Biochem. J..

[36] Willows R., Navaratnam N., Lima A., Read J., Carling D. (2017). Effect of different γ-subunit isoforms on the regulation of AMPK. Biochem. J..

[37] Philip B., Kokalaki E., Mekkaoui L., Thomas S., Straathof K., Flutter B. (2014). A highly compact epitope-based marker/suicide gene for easier and safer T-cell therapy. Blood.

[38] Martin O.J., Lai L., Soundarapandian M.M., Leone T.C., Zorzano A., Keller M.P. (2014). A role for peroxisome proliferator-activated receptor γ coactivator-1 in the control of mitochondrial dynamics during postnatal cardiac growth. Circ. Res..

[39] Ros S., Schulze A. (2013). Balancing glycolytic flux: the role of 6-phosphofructo-2-kinase/fructose 2,6-bisphosphatases in cancer metabolism. Cancer Metab..

[40] Liemburg-Apers D.C., Wagenaars J.A.L., Smeitink J.A.M., Willems P.H.G.M., Koopman W.J.H. (2016). Acute stimulation of glucose influx upon mitoenergetic dysfunction requires LKB1, AMPK, Sirt2 and mTOR-RAPTOR. J. Cell Sci..

[41] Kurth-Kraczek E.J., Hirshman M.F., Goodyear L.J., Winder W.W. (1999). 5’ AMP-activated protein kinase activation causes GLUT4 translocation in skeletal muscle. Diabetes.

[42] Brown R.A., Byersdorfer C.A. (2020). Metabolic pathways in alloreactive T cells. Front. Immunol..

[43] Gwinn D.M., Shackelford D.B., Egan D.F., Mihaylova M.M., Mery A., Vasquez D.S. (2008). AMPK phosphorylation of raptor mediates a metabolic checkpoint. Mol. Cell.

[44] Dunsford L.S., Thoirs R.H., Rathbone E., Patakas A., Tan S.-L. (2020). Methods in Pharmacology and Toxicology.

[45] Scharping N.E., Rivadeneira D.B., Menk A.V., Vignali P.D.A., Ford B.R., Rittenhouse N.L. (2021). Mitochondrial stress induced by continuous stimulation under hypoxia rapidly drives T cell exhaustion. Nat. Immunol..

[46] Dwyer C.J., Arhontoulis D.C., Rangel Rivera G.O., Knochelmann H.M., Smith A.S., Wyatt M.M. (2020). *Ex vivo* blockade of PI3K gamma or delta signaling enhances the antitumor potency of adoptively transferred CD8+ T cells. Eur. J. Immunol..

[47] Hermans D., Gautam S., García-Cañaveras J.C., Gromer D., Mitra S., Spolski R. (2020). Lactate dehydrogenase inhibition synergizes with IL-21 to promote CD8+ T cell stemness and antitumor immunity. Proc. Natl. Acad. Sci. U. S. A..

[48] Gong W., Hoffmann J.-M., Stock S., Wang L., Liu Y., Schubert M.-L. (2019). Comparison of IL-2 vs IL-7/IL-15 for the generation of NY-ESO-1-specific T cells. Cancer Immunol. Immunother..

[49] Cieri N., Camisa B., Cocchiarella F., Forcato M., Oliveira G., Provasi E. (2013). IL-7 and IL-15 instruct the generation of human memory stem T cells from naive precursors. Blood.

[50] Nelson M.H., Knochelmann H.M., Bailey S.R., Huff L.W., Bowers J.S., Majchrzak-Kuligowska K. (2020). Identification of human CD4+ T cell populations with distinct antitumor activity. Sci. Adv..

[51] Araki K., Ahmed R. (2013). AMPK: a metabolic switch for CD8+ T-cell memory. Eur. J. Immunol..

[52] Aamir S., Anwar M.Y., Khalid F., Khan S.I., Ali M.A., Khattak Z.E. (2020). Systematic review and meta-analysis of CD19-specific CAR-T cell therapy in relapsed/refractory acute lymphoblastic leukemia in the pediatric and young adult population: safety and efficacy outcomes. Clin. Lymphoma Myeloma Leuk..

[53] Guo Y., Xie Y.-Q., Gao M., Zhao Y., Franco F., Wenes M. (2021). Metabolic reprogramming of terminally exhausted CD8+ T cells by IL-10 enhances anti-tumor immunity. Nat. Immunol..

[54] Laderoute K.R., Amin K., Calaoagan J.M., Knapp M., Le T., Orduna J. (2006). 5’-AMP-activated protein kinase (AMPK) is induced by low-oxygen and glucose deprivation conditions found in solid-tumor microenvironments. Mol. Cell Biol..

[55] Zhang Y., Wang Y., Xu J., Tian F., Hu S., Chen Y. (2019). Melatonin attenuates myocardial ischemia-reperfusion injury *via* improving mitochondrial fusion/mitophagy and activating the AMPK-OPA1 signaling pathways. J. Pineal Res..

[56] Mayer K.A., Smole U., Zhu C., Derdak S., Minervina A.A., Salnikova M. (2021). The energy sensor AMPK orchestrates metabolic and translational adaptation in expanding T helper cells. FASEB J..

[57] Pandit M., Timilshina M., Gu Y., Acharya S., Chung Y., Seo S.-U. (2022). AMPK suppresses Th2 cell responses by repressing mTORC2. Exp. Mol. Med..

[58] Dai W., Xu Y., Mo S., Li Q., Yu J., Wang R. (2020). GLUT3 induced by AMPK/CREB1 axis is key for withstanding energy stress and augments the efficacy of current colorectal cancer therapies. Signal. Transduct. Target. Ther..

[59] Pokhrel R.H., Kang B., Timilshina M., Chang J.-H. (2022). AMPK amplifies IL2-STAT5 signaling to maintain stability of regulatory T cells in aged mice. Int. J. Mol. Sci..

[60] Holczer M., Hajdú B., Lőrincz T., Szarka A., Bánhegyi G., Kapuy O. (2019). A double negative feedback loop between mTORC1 and AMPK kinases guarantees precise autophagy induction upon cellular stress. Int. J. Mol. Sci..

[61] Afinanisa Q., Cho M.K., Seong H.-A. (2021). AMPK localization: a key to differential energy regulation. Int. J. Mol. Sci..

[62] Chauhan A.S., Zhuang L., Gan B. (2020). Spatial control of AMPK signaling at subcellular compartments. Crit. Rev. Biochem. Mol. Biol..

[63] Hardy R., Pryde K. (2020). AMPK and the PI3K-mTORC2-AKT Axis shape the mitochondrial integrated-stress-response. SSRN J..

[64] Han F., Li C.-F., Cai Z., Zhang X., Jin G., Zhang W.-N. (2018). The critical role of AMPK in driving Akt activation under stress, tumorigenesis and drug resistance. Nat. Commun..

[65] Bantug G.R., Fischer M., Grählert J., Balmer M.L., Unterstab G., Develioglu L. (2018). Mitochondria-endoplasmic reticulum contact sites function as immunometabolic hubs that orchestrate the rapid recall response of memory CD8+ T cells. Immunity.

[66] Lu Y., Hippen K.L., Lemire A.L., Gu J., Wang W., Ni X. (2016). miR-146b antagomir-treated human Tregs acquire increased GVHD inhibitory potency. Blood.

[67] Magee J.A., Ikenoue T., Nakada D., Lee J.Y., Guan K.-L., Morrison S.J. (2012). Temporal changes in PTEN and mTORC2 regulation of hematopoietic stem cell self-renewal and leukemia suppression. Cell Stem Cell.

