# Supplementary material for: Overexpression of AMPKγ2 increases AMPK signaling to augment human T cell metabolism and function
Source: J Biol Chem. 2023 Nov 22;300(1):105488. doi: 10.1016/j.jbc.2023.105488 (PMC10825059; doi:10.1016/j.jbc.2023.105488)
Supplement: Supporting information [file mmc1.pdf]

Supporting Information

Title: Overexpression of AMPKγ2 increases AMPK signaling to augment human T cell metabolism and function

Authors: Erica L. Braverman[1], Margaret A. McQuaid[1], Herbert Schuler[1], Mengtao Qin[1], Sophia Hani[2], Keli Hippen[2], Darlene A. Monlish[1], Andrea K. Dobbs[1], Manda J Ramsey[1], Felcia Kemp[1], Christopher Wittmann[1], Archana Ramgopal[1], Harrison Brown[1], Bruce Blazar[2], and Craig A. Byersdorfer*[a]

Contents

Figure S1-S4

Table S1-S3

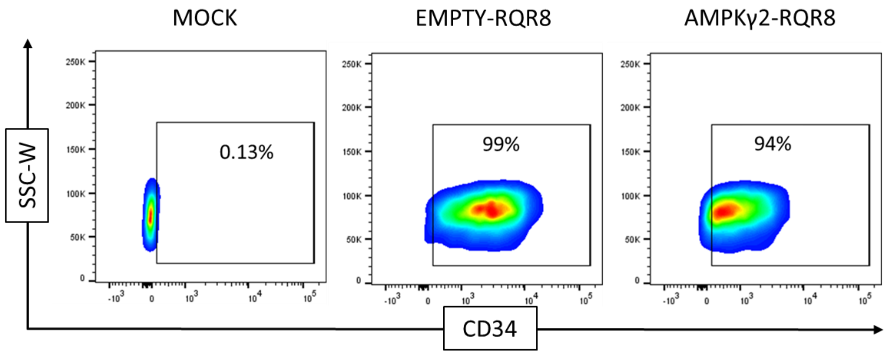

Figure S1. AMPKγ2 overexpression in human T cells. Primary human T cells were mock transduced or transduced with AMPKγ2 or Empty plasmids. Expression was verified by flow cytometry for the CD34 motif of the RQR8 construct on day 5 of culture.

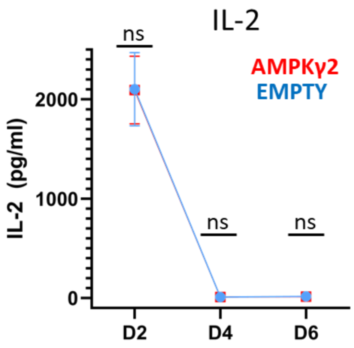

Figure S2. AMPK-transduction does not decrease IL-2 production. AMPKγ2- versus Empty-transduced cells were placed into an *in vitro* exhaustion protocol where they were re-stimulated with CD3/CD28 Dynabeads every 48 hours over 6 days. L-2 was measured at each timepoint by ELISA.

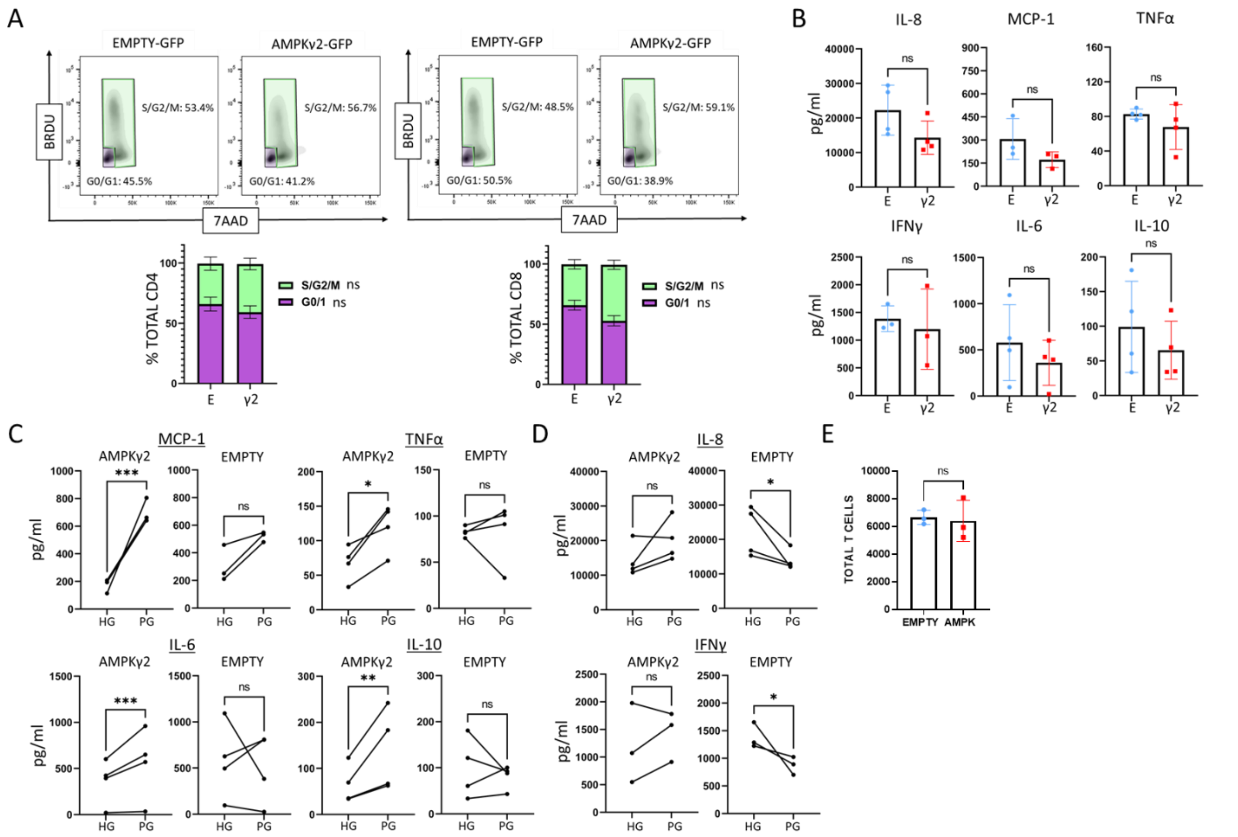

Figure S3. AMPKγ2 transduced cells function similar to control cells in high glucose media but increase cytokine production when challenged in physiologic glucose. (A) AMPKγ2- and Empty-transduced T cells were cultured for 9 days then re-stimulated in 11 mM glucose for 72 hours, incubated with BrdU for 2 hours, counter-stained with 7AAD, and run on the flow cytometry for cell cycle analysis. **(B-D)** AMPK- and Empty-transduced T cells were cultured for 9 days then plated against allogeneic non-T cell APCs in media containing either 11 mM (high glucose (HG)) or 5.5 mM (physiologic glucose (PG)) for 72 hours. Media was harvested and assessed for cytokine expression by LegendPlex analysis. Cytokine levels were first compared between AMPK- and Empty-transduced T cells in HG media **(B)** and then within the same treatment group in HG versus PG conditions **(C-D)**. AMPKγ2-transduced cells increased **(C)** or maintained **(D)** concentrations of inflammatory cytokines which was in contrast to the behavior seen in control cells. To assess cell counts, MLRs were harvested after 72 hours, and T cell counts assessed using CountBright Counting Beads (Thermo) **(E).** Flow plots represent data from 2-4 independent donors, while graphs are composites of all data. Bar graphs in (A) represent 3 independent donors. *p<0.05, **p<0.01, ***p<0.001 by paired Student's T test

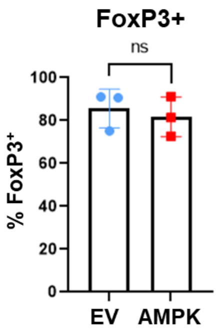

Figure S4. AMPKγ2 overexpression does not impact FoxP3 levels in Treg cells. Human Treg cells were sorted, transduced with AMPKγ2 or EV-lentiviral vectors, and expanded in IL-2. Intracellular FoxP3 expression was assessed by intracellular staining and flow cytometry on Day 7, where data represent results obtained from three independent human donors.

Supporting tables

Table S1 – Antibodies for Immunoblot analysis

Antigen	Company	Clone	Catalog #
**Phospho-ACC**	*Cell Signaling*	D7D11	11818S
**ACC**	*Cell Signaling*	C83B10	3676S
**Phospho-ULK-1**	*Cell Signaling*	D1H4	5869S
**ULK-1**	*Cell Signaling*	D8H5	8054S
**Opa1**	*Cell Signaling*	D6U6N	80471S
**Mfn1**	*Cell Signaling*	D6E2S	14739S
**Phospho-AMPK**	*Cell Signaling*	T172	2535S
**AMPK**	*Cell Signaling*	F6	2793S
**Beta Actin**	*Cell Signaling*	13E5	4970S
**PGC1a**	*Cell Signaling*	3G6	2178S

Abbreviations: ACC Acetyl CoA Carboxylase, AMPK AMP-activated protein kinase, ULK1 Unc51-like kinase 1

Table S2 – Antibodies and reagents for flow cytometry

Antibody	Conjugate	Company	clone
PD1	Per-CP	BIOLEGEND	EH12.2H7
CD8	EF780	INVITROGEN	SK1
LAG3	PE	BIOLEGEND	11C3C65
CD4	PeCY7, APC, PE, eF450	INVITROGEN	RPA-T4
CD25	BV711, BV605	BIOLEGEND	BC96
TIM3	BV605	BIOLEGEND	F38-2E2
CD45RA	APC	INVITROGEN	HI100
CD62L	BV605	BIOLEGEND	DREG-56
IFNG	EF780	INVITROGEN	4SB3
IL10	PeCY7	INVITROGEN	JES3-9D7
IL2	BV711	BD BIOSCIENCES	5344.111
TNFα	BV605	BIOLEGEND	MAb11
PS6	Per-CP	INVITROGEN	cupk43k
CD4	Pac Blue	BIOLEGEND	SK3
CD8	BV711 APC	BD BIOSCIENCES	RPA-T8
CD8	Per-CP	BD BIOSCIENCES	SK1
P4EBP1	PE	INVITROGEN	V3NTY24
FOXP3	Pac Blue	BIOLEGEND	eBioRDR5
CD127	Per-CP	INVITROGEN	206D
CD34	PE	R&D SYSTEMS	FAB7227P
Additional Flow Reagents			
**Reagent**	**Channel**	**Company**	
Vybrant™ CFDA SE Cell Tracer Kit	FITC (488nm)	INVITROGEN	
Zombie Aqua	BV510	BIOLEGEND	
CountBright Beads		Thermo Fisher	

Table S3 – Primers for qRT-PCR

Target	Forward	Reverse
**Human IL-4**	CCGTAACAGACATCTTTGCTGCC	GAGTGTCCTTCTCATGGTGGCT
**Human IL-5**	GGAATAGGCACACTGGAGAGTC	CTCTCCGTCTTTCTTCTCCACAC
